# Living with Hypertension: An Investigation of Illness Perception from a Primary Care Perspective

**DOI:** 10.3390/healthcare13162032

**Published:** 2025-08-17

**Authors:** Handan Duman, Latife Merve Yildiz

**Affiliations:** 1 Department of Family Medicine, Recep Tayyip Erdogan University Faculty of Medicine, 53100 Rize, Turkey; handan.duman@erdogan.edu.tr; 2 Department of Family Medicine, Ordu University Faculty of Medicine, 52200 Ordu, Turkey

**Keywords:** hypertension, illness perception, primary care, treatment adherence, health literacy

## Abstract

**Background:** This study aimed to assess the illness perceptions of patients diagnosed with hypertension from a primary care perspective. It also sought to identify the sociodemographic and clinical factors associated with illness perception. **Methods:** A cross-sectional descriptive study was conducted between February and March 2025, involving 116 hypertensive patients who attended family medicine outpatient clinics at Rize Training and Research Hospital, Turkey. Data were collected using a sociodemographic questionnaire and the Brief Illness Perception Questionnaire (BIPQ). Nonparametric tests, including the Mann–Whitney U test, Kruskal–Wallis H test with Dunn’s post hoc analysis, and Spearman’s correlation analysis, were employed to evaluate the data. **Results:** The mean age of the participants was 69.01 ± 6.07 years, with 76.7% of the participants aged over 65 years. The median total BIPQ score was 47.0, indicating a moderate illness perception. A significant negative correlation was observed between age and the total BIPQ score (Rho = −0.443, *p* < 0.001). Higher illness perception levels were significantly associated with lower educational attainment, shorter duration of antihypertensive treatment, and attribution of hypertension to stress, genetic predisposition, diet, and occupational factors (*p* < 0.05). **Conclusions:** Illness perceptions among patients with hypertension are shaped by various sociodemographic and clinical determinants. Enhancing awareness of these perceptions in primary care may support improved treatment adherence and better health outcomes. Interventions that strengthen health literacy and offer psychosocial support may contribute to more effective hypertension management.

## 1. Introduction

Hypertension is defined as a persistent elevation of arterial blood pressure above normal levels. According to the 2013 guidelines published by the European Society of Cardiology (ESC) and the European Society of Hypertension (ESH), hypertension is characterized by a systolic blood pressure (SBP) exceeding 140 mmHg and/or a diastolic blood pressure (DBP) exceeding 90 mmHg [1].

Hypertension is a chronic condition that affects approximately one billion people worldwide and leads to serious health complications. In 2000, the global prevalence of hypertension was reported to be 26.4%, with projections indicating a rise to 29.2% by 2025 [2]. According to the World Health Organization (WHO), cardiovascular diseases account for 17.9 million deaths globally each year, and 31% of these deaths are attributed to hypertension [3]. Recent global analyses reveal an even higher burden of hypertension. A 2021 WHO report indicated that 1.28 billion adults aged 30 to 79 years are currently living with hypertension, and approximately 46% of them are unaware of their condition [4]. Furthermore, nearly three-quarters of hypertensive individuals live in low- and middle-income countries [4].

In Turkey, the Turkish Adult Heart Disease and Risk Factors Survey (TEKHARF) reported a prevalence of 33.7% [5]. The Turkish Hypertension Prevalence Study (PatenT), conducted in 2003, found a prevalence of 31.8%, and the PatenT 2 study in 2012 reported 30.3% [6,7]. In the Metabolic Syndrome Prevalence Study in Turkish Adults (METSAR), the prevalence was reported to be 41.7% [8]. Additionally, 2017 data from the Turkish Statistical Institute (TÜİK) indicated that 39.7% of all deaths in Turkey were due to cardiovascular diseases, with 8.9% specifically related to hypertension [9]. Another study conducted in Turkey found hypertension prevalence to be 32.3% in women, 28.4% in men, and 30.3% overall [10]. The same study reported hypertension awareness rates of 66.9% among women, 40.6% among men, and 54.7% overall [10]. In light of these findings, the concept of illness perception has gained growing importance among patients with hypertension, as in many other chronic diseases.

Hypertension frequently coexists with other metabolic disorders, particularly diabetes mellitus. More than 50% of individuals with type 2 diabetes also have hypertension, and this comorbidity substantially increases cardiovascular mortality risk and complicates disease management [11]. One study reported that approximately one-third of hypertensive individuals also had diabetes as a comorbid condition [12]. The shared pathophysiological mechanisms between hypertension and diabetes—including insulin resistance, obesity, and chronic inflammation—may influence patients’ illness perceptions directly [13].

Illness perception refers to an individual’s cognitive and emotional responses to their disease [14]. Factors such as physical and mental well-being, cultural values, and quality of life significantly influence this perception. It encompasses cognitive dimensions—including identity, timeline, consequences, treatment control, and coherence—as well as emotional reactions such as stress and anger [14]. Illness perceptions may vary among individuals with the same diagnosis, and even healthcare professionals may have differing interpretations of the same condition.

Illness perception is a significant factor in the management of hypertension [15]. Ross et al. [16] emphasized that, beyond pharmacological treatment, illness perception plays a crucial role in the effective management of hypertension. Although research on illness perception among hypertensive patients in Turkey remains limited, Oğuz et al. [17] found that patients exhibited high illness perception scores and tended to perceive their condition as chronic. Similarly, Norfazilah et al. [18] reported high levels of illness perception among hypertensive patients in Malaysia, with family history and anxiety identified as influential factors. A meta-analysis by Kılıçkap et al. [19] revealed that nearly 11 million individuals in Turkey have uncontrolled hypertension, underlining the need for more research on illness perception in this population.

In primary healthcare settings, patients’ illness perceptions have a direct impact on treatment adherence and healthcare processes [20]. In chronic diseases such as hypertension, patients’ understanding of their illness, disease-related awareness, knowledge levels, and personal experiences significantly shape their coping mechanisms and adaptation processes [16]. Illness perception not only influences physical, emotional, and psychosocial responses but also plays a critical role in treatment adherence and quality of life [21]. Negative perceptions may lead to nonadherence, heightened anxiety, and delayed recovery, whereas adequate information and positive perceptions can contribute to more effective disease management and enhanced quality of life [22].

In this study, the Brief Illness Perception Questionnaire (BIPQ) was used to evaluate illness perception among patients with hypertension in a primary care setting. This research aims to contribute to a deeper understanding of illness perception among hypertensive patients within the scope of primary healthcare, particularly family medicine, and to provide a foundation for developing more effective treatment and care strategies.

## 2. Materials and Methods

### 2.1. Aim and Study Design

This study was conducted to determine the illness perception levels of patients with hypertension and to identify the factors influencing their illness perception. A descriptive cross-sectional study design was employed.

### 2.2. Population and Sample

The study was conducted over a two-month period between February and March 2025, at the Family Medicine Outpatient Clinics of Rize Training and Research Hospital. During this period, a total of 413 patients presented to the outpatient clinic. Among them, 116 patients who met the inclusion criteria and voluntarily agreed to participate were included in the study.

Participants were selected using a convenience sampling method, whereby individuals meeting the inclusion criteria and presenting during the study period were non-randomly enrolled. This approach involves the inclusion of individuals who are accessible to the researcher and willing to participate, without randomization.

The sample size was calculated using G*Power version 3.1.9.4 software (Heinrich Heine University, Düsseldorf, Germany). Based on parameters of the chi-square goodness-of-fit test—effect size: 0.35, type I error (α): 0.05, power (1–β): 0.80, and degrees of freedom: 4—the minimum required sample size was determined to be 98 participants. These parameters are consistent with widely accepted conventions in behavioral and social sciences for detecting moderate effect sizes in cross-sectional studies [23].

Inclusion criteria were being aged 18 years or older; having received a hypertension diagnosis at least six months prior; being able to communicate effectively; having been informed about the study; and providing voluntary consent to participate.

### 2.3. Data Collection Tools and Procedure

Data were collected using two instruments: a sociodemographic information form developed based on a literature review and the BIPQ. The sociodemographic form included items regarding participants’ demographic characteristics (e.g., age, sex, educational level, occupation) and health-related variables (e.g., height, weight, smoking and alcohol use, comorbidities, time since diagnosis, polypharmacy status).

All data were collected through face-to-face interviews conducted by the researcher. Completion of the sociodemographic form and BIPQ took approximately 20–30 min per participant.

### 2.4. Brief Illness Perception Questionnaire (BIPQ)

The BIPQ, developed by Broadbent et al. [21], is a brief measure designed to assess individuals’ cognitive and emotional representations of illness. It consists of eight items rated on a 0–10 scale. Higher scores on items 3 (personal control), 4 (treatment control), and 7 (coherence) reflect a more positive perception of the illness. In contrast, higher scores on items 1 (consequences), 2 (timeline), 5 (illness comprehensibility), 6 (concern), and 8 (emotional representation) indicate a more negative illness perception. Items 1, 2, 5, 6, and 8 are reverse scored. That is, for these items, higher raw scores indicate a more negative illness perception, whereas for items 3, 4, and 7, higher scores denote a more positive perception. The total BIPQ score ranges from 0 to 80, with higher scores indicating a more negative overall perception of illness [21].

The Turkish version of the BIPQ has been validated and demonstrated strong psychometric properties. The scale is unidimensional and provides reliable assessment across a broad scoring range. In the Turkish validation study, concurrent validity coefficients ranged from 0.843 to 0.854; Cronbach’s alpha coefficient was 0.944; and the intraclass correlation coefficient (ICC) for test–retest reliability was 0.987 [24].

### 2.5. Statistical Analysis

All statistical analyses were performed using IBM SPSS version 25.0 (IBM Corp., Armonk, NY, USA). Continuous variables are presented as median (IQR) and mean ± standard deviation (SD), while categorical variables are presented as counts (n) and percentages (%).

The normality of continuous variables was assessed using the Kolmogorov–Smirnov test and skewness/kurtosis values. Since *p*-values from the Kolmogorov–Smirnov test were <0.05 and skewness/kurtosis standardized values exceeded ±3.29, nonparametric tests were used for further analysis [25,26].

The relationship between the BIPQ scores and continuous variables was assessed using Spearman’s correlation analysis. Comparisons between two groups were made using the Mann–Whitney U test, and comparisons among three or more groups were made using the Kruskal–Wallis H test. In cases where the Kruskal–Wallis test indicated significant differences, Dunn’s post hoc test was performed to determine pairwise differences. A *p*-value < 0.05 was considered statistically significant.

## 3. Results

This section presents the statistical findings obtained from the Brief Illness Perception Questionnaire (BIPQ). First, the sociodemographic and health-related characteristics of the participants are described. Then, total and subdimension BIPQ scores are compared across various groups (Table 1).

The mean age of the participants was 69.01 ± 6.07 years, and 76.7% (*n* = 89) were over the age of 65. Of the participants, 55.2% were female, 51.7% were university graduates, and 37.9% were retired. A total of 72.4% were overweight, 34.5% had no comorbid conditions, 62.1% received information about their disease from a physician, 37.9% had initiated antihypertensive treatment 6–10 years ago, and 24.1% were undergoing polypharmacy.

The mean total and subdimension BIPQ scores are presented in Table 2. The highest mean score was observed in the timeline subdimension (8.79 ± 1.52), while the lowest was observed in the treatment control subdimension (3.72 ± 1.67). A higher score in the timeline subdimension reflects a perception that the illness will persist for a long time, whereas a lower score in treatment control indicates that individuals perceive their ability to manage treatment as limited.

A significant negative correlation was identified between age and total BIPQ score (Rho = −0.443, *p* < 0.001). Since higher total scores reflect a more negative illness perception, this finding indicates that participants aged over 65 tend to perceive their illness less negatively (Table 3).

Differences in the total BIPQ scores according to participants’ age, sex, educational status, and other sociodemographic characteristics are presented in detail in Table 4.

When comparing total BIPQ scores by age group, participants aged ≤65 years had higher scores than those aged >65 years (Z = −3.487, *p* < 0.001), indicating a more negative perception of illness in the younger age group.

A significant difference was also found in total BIPQ scores according to educational status (KW = 20.210, *p* < 0.001). Illiterate participants had higher total scores, reflecting a more negative perception. Post hoc analysis revealed significant differences between university graduates and those with a high school education or below (*p* = 0.037), and between university graduates and literate participants (*p* = 0.001).

Occupational status was also significantly associated with total BIPQ scores (KW = 30.202, *p* < 0.001). Officers had higher total scores than other groups, indicating a more negative illness perception in this category. Post hoc analysis revealed significant differences between unemployed individuals and workers (*p* < 0.001), workers and officers (*p* = 0.001), workers and retirees (*p* = 0.001), and retirees and unemployed individuals (*p* = 0.002).

Regarding the duration of antihypertensive use, a significant difference was found (KW = 6.757, *p* = 0.034). Participants who had been using antihypertensives for 2–5 years had higher total BIPQ scores compared to those treated for more than 10 years (post hoc *p* = 0.029), reflecting a more negative illness perception among individuals with shorter treatment durations.

A significant difference in total BIPQ scores was observed according to participants’ attribution of their illness to stress (KW = 9.772, *p* = 0.008). Those who responded “definitely yes” had higher scores, indicating more negative illness perceptions compared to those who responded “undecided” (*p* = 0.019) and “yes” (*p* = 0.026).

A similar finding was noted in the attribution of illness to hereditary factors (KW = 10.791, *p* = 0.005). Participants who responded “yes” had higher scores than those who were “undecided” (*p* = 0.007), indicating a more negative illness perception in the former group.

Regarding diet attribution, a significant difference in total BIPQ scores was found (KW = 31.037, *p* < 0.001). Participants who responded “definitely yes” had higher scores, reflecting more negative perceptions of illness compared to those who responded “undecided” (*p* < 0.001 and *p* = 0.002, respectively).

Finally, a significant difference in total BIPQ scores was observed in relation to attribution of illness to occupational factors (KW = 19.072, *p* = 0.001). Participants who responded “definitely yes” had higher scores than those who responded “definitely no” (*p* = 0.006), again indicating more negative illness perceptions in this group.

## 4. Discussion

Illness perception is a critical concept directly associated with treatment adherence and coping strategies in the management of chronic diseases. This study aimed to examine the illness perceptions of patients diagnosed with hypertension and the factors influencing these perceptions. A review of the literature reveals that illness perception has been evaluated across various chronic conditions, and the BIPQ is commonly used. However, studies focusing specifically on patients with hypertension remain limited.

In a study conducted by Karadağ et al. [27] with patients diagnosed with hypertension, the mean score of the timeline subscale was reported as 18.89 ± 3.25. Similarly, Oğuz et al. [17] reported a score of 24.92 ± 5.58 for the same subscale. Both studies reported higher scores compared to our findings. These differences may be explained by variations in sample size, demographic and clinical characteristics of the study population, or methodological differences. A strong perception of chronicity may lead individuals to perceive the illness as uncontrollable and inevitable, which has been associated with depressive mood, feelings of helplessness, and poor treatment adherence [28].

The low perception of treatment control in our sample may reflect a lack of confidence in the treatment’s effectiveness or skepticism toward the healthcare system, which can negatively affect adherence and clinical outcomes. Karadağ et al. [27] reported a significantly higher treatment control score (17.32 ± 3.32). This discrepancy may be due to differences in healthcare access, educational background, or treatment-related attitudes between study populations. Similarly, a study involving patients with coronary artery disease also reported low treatment control perceptions and limited trust in the treatment process [29]. Individual health literacy-based training sessions conducted in primary care have been shown to improve this perception positively [30]. Additionally, motivational interviewing techniques and self-efficacy-enhancing counseling approaches may strengthen patients’ perception of treatment control [31].

According to BIPQ scoring, this finding suggests that patients may have difficulties in understanding their illness or may perceive it as complex. This highlights the need to reinforce health education and information delivery processes. Especially among elderly or individuals with limited literacy skills, simplified written materials and guided one-on-one interviews have been proven effective in improving illness comprehension [32].

The concern and emotional representation subscale scores suggest that patients with hypertension experience notable emotional distress and anxiety related to their illness. In comparison, the emotional representation score reported by Karadağ et al. [27] was significantly higher (21.31 ± 5.74). This discrepancy may be explained by sample characteristics, sociocultural factors of the research setting, or variability in coping strategies. In the literature, stress management training, relaxation techniques, and group-based psychoeducation programs have been shown to be effective in reducing emotional burden [33].

Our finding of a negative correlation suggests that illness perception becomes more positive with increasing age. In other words, older individuals tend to have a better understanding of their illness and adopt more constructive coping attitudes. This could imply that aging individuals gradually accept their health conditions and develop improved self-management skills. It is recommended that this trend be supported through continuous educational interventions tailored to older adults in primary care settings [34].

The observed difference suggests that individuals with lower educational levels tend to perceive their illness more negatively, while university graduates demonstrate more adaptive perceptions and understanding. Higher education likely enhances access to health information and coping strategies. Similarly, a study from Iran found that lower educational status was associated with more negative illness perceptions, which adversely affected treatment adherence [35]. Targeted patient education and individualized health counseling programs implemented in primary care have been supported in the literature as effective strategies to improve illness perception among low-education populations [36].

The finding that civil servants perceive their illness more negatively may reflect the influence of occupational conditions, work-related stress, and access to professional information. Studies have highlighted that the nature of one’s job and occupational security shape how individuals interpret and react to illness [37]. Sluiter and Frings-Dresen [38] also reported a relationship between illness perception and occupational background. In particular, positions associated with high cognitive load and job stress have been shown to contribute to perceiving illnesses as more threatening [39].

The literature suggests that individuals newly initiated on treatment often perceive their illness as more complex and distressing; however, this perception may normalize over time. Among patients receiving long-term treatment, improvements in self-management and consistent follow-up care have been shown to reduce the psychological burden of illness [40].

Individuals who attribute their illness to stress, genetics, diet, or occupation tend to perceive their illness more negatively or feel its impact more strongly. Previous studies support this observation. For example, in patients with inflammatory bowel disease, stress has been shown to significantly affect disease activity, quality of life, and psychological well-being [41]. Similarly, a study on early-stage lung cancer patients found an association between stress and illness perception [42]. Regarding genetic attributions, individuals who emphasize hereditary causes often perceive their condition as more serious and inevitable, which can increase anxiety levels [43]. A study conducted in 2016 with hypertensive patients also reported that attributing the illness to genetic causes negatively influenced illness perception [44]. These findings highlight the important role of genetic beliefs in shaping illness perception and psychosocial adaptation.

Individuals who perceive diet as a cause of their illness may experience increased emotional burden due to the need for dietary changes or restrictions. This perception may lead to a more negative view of their condition. A study conducted among patients with diabetes demonstrated that attributing illness to diet influenced illness perception and led to increased responsibility in managing health [45]. Similarly, a study involving 84 individuals showed that work-related stress and occupational disease significantly influenced illness perception. Stress and pressure in the work environment have been linked to more negative views of chronic illness, reduced treatment adherence, and worsened disease progression. These findings are consistent with our study’s observation regarding the influence of work-related factors on illness perception [46]. 

### Limitations

This study has several limitations. Its cross-sectional design does not allow for causal inferences. Since the research was conducted in a single-center using a convenience sampling method, the representativeness of the sample for the general hypertensive population is limited. Additionally, no data were collected regarding the stage or clinical severity of hypertension. The relatively small sample size may also reduce the power of subgroup analyses. Furthermore, a multivariate model including multiple explanatory variables was not conducted due to the limited sample size, which may have restricted the ability to control for potential confounding factors. Future multi-center and longitudinal studies are recommended to support these findings.

## 5. Conclusions

This study evaluated the illness perception levels of individuals diagnosed with hypertension and the sociodemographic and clinical factors influencing these perceptions. The overall illness perception among participants was found to be at a moderate level. Illness perception scores were significantly associated with variables such as age, educational level, occupational status, and duration of antihypertensive medication use. Additionally, participants who attributed the cause of their illness to stress, genetic predisposition, diet, or occupational factors exhibited higher illness perception scores. These findings indicate that patients’ perceptions of hypertension are shaped by a range of individual and contextual factors. The results suggest that structured approaches to assess illness perception may be necessary within the scope of primary healthcare services.

## Figures and Tables

**Table 1:** Frequency distribution of sociodemographic and various clinical variables of the participants (*n* = 116).

Variables	*n* or Median (Min–Max)	% or Mean ± SD
Age (years)	67.0 (33.0–84.0)	69.01 ± 6.07
Age Group		
≤65 years	27	23.3%
>65 years	89	76.7%
Gender		
Female	64	55.2%
Male	52	44.8%
Educational Status		
Illiterate	8	6.9%
Literate	44	37.9%
High School or Below	4	3.4%
University Graduate	60	51.7%
Occupation		
Worker	28	24.1%
Officer	12	10.3%
Retired	44	37.9%
Unemployed	32	27.6%
Height (cm)	167.0 (160.0–185.0)	167.96 ± 5.70
Weight (kg)	80.0 (70.0–120.0)	82.34 ± 11.11
BMI	28.0 (25.0–39.0)	29.14 ± 3.19
BMI Group		
Overweight	84	72.4%
Obesity Class 1	20	17.2%
Obesity Class 2	12	10.3%
Comorbidities		
Diabetes Mellitus (DM)	12	10.3%
Coronary Artery Disease (CAD)	32	27.6%
Other	32	27.6%
None	40	34.5%
Source of Information about Disease		
Doctor	72	62.1%
Nurse/Healthcare Personnel	44	37.9%
Duration since Antihypertensive Initiation		
2–5 years	40	34.5%
6–10 years	44	37.9%
>10 years	32	27.6%
Polypharmacy		
Yes	28	24.1%
No	88	75.9%
Perceived Cause of Disease		
Stress	72	62.1%
Occupation	44	37.9%
Stress Attribution		
Undecided	20	17.2%
Yes	60	51.7%
Definitely Yes	36	31.0%
Genetic Attribution		
No	4	3.4%
Undecided	80	69.0%
Yes	32	27.6%
Diet Attribution		
No	56	48.3%
Undecided	24	20.7%
Yes	32	27.6%
Definitely Yes	4	3.4%
Occupation Attribution		
Definitely No	8	6.9%
No	52	44.8%
Undecided	32	27.6%
Yes	16	13.8%
Definitely Yes	8	6.9%
Aging Attribution		
No	28	24.1%
Undecided	72	62.1%
Yes	16	13.8%
Alcohol Use		
Undecided	52	44.8%
Yes	64	55.2%
Tobacco Use		
Undecided	52	44.8%
Yes	64	55.2%

*n* = number; Min = minimum; Max = maximum; Mean = average; SD = standard deviation.

**Table 2:** Distribution of the total and subdimension scores of the BIPQ (*n* = 116).

Variables	Median	Min	Max	Mean	SD
BIPQ Total	47.0	38.00	68.00	48.66	7.41
1. Consequences	7.0	3.00	10.00	7.07	2.11
2. Timeline	9.0	5.00	10.00	8.79	1.52
3. Personal Control	5.0	1.00	9.00	5.38	1.98
4. Treatment Control	3.0	2.00	7.00	3.72	1.67
5. Illness Comprehensibility	8.0	4.00	10.00	7.28	1.83
6. Concern	6.0	2.00	10.00	6.14	2.41
7. Coherence	5.0	1.00	7.00	4.21	1.70
8. Emotional Representation	6.0	3.00	10.00	6.07	1.67

*n* = number; Min = minimum; Max = maximum; Mean = average; SD = standard deviation.

**Table 3:** Correlation analysis between the total BIPQ score, age, and BMI.

	Age (years)	BMI (kg/m^2^)
BIPQ Total Score	Rho = −0.443	Rho = −0.015
	*p* < 0.001	*p* = 0.872

Spearman’s correlation test was used. Rho: Spearman’s correlation coefficient. *p* < 0.05.

**Table 4:** Comparison of the total BIPQ scores according to the sociodemographic and clinical characteristics of the patients.

Variables	Groups	*n*	Median (IQR)	Mean ± SD	Z/KW	*p*	Post Hoc
Age Group	≤65 years	27	55.0 (11.0)	52.96 ± 7.98	−3.487	<0.001	
	>65 years	89	46.0 (5.0)	47.35 ± 6.75			
Gender	Female	64	46.5 (12.5)	48.63 ± 7.51	−0.178	0.859	
	Male	52	49.0 (6.0)	48.69 ± 7.36			
Educational Status	Illiterate	8	54.5 (21.0)	54.50 ± 11.22	20.210	<0.001	3 < 4, 2 < 4
	Literate	44	45.0 (3.0)	45.45 ± 5.11			
	High School or Below	4	43.0 (0.0)	43.00 ± 0.01			
	University Graduate	60	49.0 (11.0)	50.60 ± 7.40			
Occupation	Worker	28	52.0 (8.0)	51.71 ± 6.57	30.202	<0.001	1 > 4, 1 < 2, 1 > 3, 2 > 3
	Officer	12	55.0 (8.0)	53.67 ± 3.55			
	Retired	44	46.0 (4.0)	47.18 ± 7.01			
	Unemployed	32	45.0 (9.0)	46.13 ± 8.10			
BMI Group	Overweight	84	47.0 (6.0)	48.43 ± 6.84	1.759	0.415	
	Class I Obesity	20	44.0 (9.0)	49.00 ± 10.78			
	Class II Obesity	12	49.0 (10.0)	49.67 ± 4.29			
Comorbidities	Diabetes Mellitus	12	45.0 (10.0)	46.33 ± 4.38	7.433	0.059	
	Coronary Artery Disease	32	47.5 (11.75)	50.75 ± 7.91			
	Other	32	47.5 (11.25)	49.75 ± 5.44			
	None	40	46.5 (11.0)	46.80 ± 8.57			
Disease-related Information	Doctor	72	47.5 (10.0)	49.56 ± 7.89	−1.142	0.254	
	Nurse/Healthcare Worker	44	47.0 (9.0)	47.18 ± 6.37			
Duration of Antihypertensive Use	2–5 years	40	48.5 (11.0)	50.60 ± 8.09	6.757	0.034	1 > 3
	6–10 years	44	48.0 (7.0)	47.36 ± 4.53			
	>10 years	32	45.0 (11.5)	48.00 ± 9.23			
Polypharmacy	Yes	28	45.0 (7.0)	48.43 ± 4.68	−0.052	0.959	
	No	88	47.5 (12.0)	48.73 ± 8.11			
Cause: Stress	Undecided	20	46.0 (3.0)	45.20 ± 3.97	9.772	0.008	3 > 1, 3 > 2
	Yes	60	46.0 (8.0)	48.07 ± 7.56			
	Definitely Yes	36	52.0 (10.0)	51.56 ± 7.71			
Cause: Genetic	No	4	45.0 (0.0)	45.00 ± 0.01	10.791	0.005	3 > 2
	Undecided	80	46.0 (12.5)	47.65 ± 7.38			
	Yes	32	50.0 (7.5)	51.63 ± 7.18			
Cause: Diet	No	56	45.5 (5.0)	46.00 ± 6.26	31.037	<0.001	2 > 1, 2 > 3
	Undecided	24	57.0 (8.0)	54.00 ± 4.79			
	Yes	32	47.0 (10.0)	48.50 ± 8.78			
	Definitely Yes	4	55.0 (0.0)	55.00 ± 0.01			
Cause: Occupation	Definitely No	8	45.0 (2.0)	45.00 ± 1.07	19.072	0.001	4 > 1
	No	52	47.0 (4.0)	47.92 ± 7.03			
	Undecided	32	45.0 (14.25)	49.50 ± 9.10			
	Yes	16	54.5 (7.25)	53.75 ± 3.53			
	Definitely Yes	8	43.5 (11.0)	43.50 ± 5.88			
Cause: Aging	No	28	48.0 (14.0)	48.14 ± 8.67	0.150	0.928	
	Undecided	72	46.0 (11.0)	48.28 ± 6.09			
	Yes	16	46.5 (18.75)	51.25 ± 10.1			
Alcohol Use	Undecided	52	47.0 (7.0)	47.77 ± 5.52	−0.401	0.688	
	Yes	64	47.0 (13.0)	49.38 ± 8.63			
Tobacco Use	Undecided	52	47.0 (7.0)	47.77 ± 5.52	−0.401	0.688	
	Yes	64	47.0 (13.0)	49.38 ± 8.63			

M = median; IQR = interquartile range; Mean = average; SD = standard deviation; Z = Mann–Whitney U test statistic; KW = Kruskal–Wallis H test statistic; Post Hoc = Dunn’s test was used for multiple comparisons.

## Data Availability

The original data presented in the study are openly available in Zenodo at https://doi.org/10.5281/zenodo.16531711.
